# Neuroactive substances specifically modulate rhythmic body contractions in the nerveless metazoon *Tethya wilhelma *(Demospongiae, Porifera)

**DOI:** 10.1186/1742-9994-3-7

**Published:** 2006-04-27

**Authors:** Kornelia Ellwanger, Michael Nickel

**Affiliations:** 1 Department of Zoology, Biological Institute, University of Stuttgart, 70550 Stuttgart, Germany

## Abstract

**Background:**

Sponges (Porifera) are nerve- and muscleless metazoa, but display coordinated motor reactions. Therefore, they represent a valuable phylum to investigate coordination systems, which evolved in a hypothetical Urmetazoon prior to the central nervous system (CNS) of later metazoa. We have chosen the contractile and locomotive species *Tethya wilhelma *(Demospongiae, Hadromerida) as a model system for our research, using quantitative analysis based on digital time lapse imaging. In order to evaluate candidate coordination pathways, we extracorporeally tested a number of chemical messengers, agonists and antagonists known from chemical signalling pathways in animals with CNS.

**Results:**

Sponge body contraction of *T. wilhelma *was induced by caffeine, glycine, serotonine, nitric oxide (NO) and extracellular cyclic adenosine monophosphate (cAMP). The induction by glycine and cAMP followed patterns varying from other substances. Induction by cAMP was delayed, while glycine lead to a bi-phasic contraction response. The frequency of the endogenous contraction rhythm of *T. wilhelma *was significantly decreased by adrenaline and NO, with the same tendency for cAMP and acetylcholine. In contrast, caffeine and glycine increased the contraction frequency. The endogenous rhythm appeared irregular during application of caffeine, adrenaline, NO and cAMP. Caffeine, glycine and NO attenuated the contraction amplitude. All effects on the endogenous rhythm were neutralised by the washout of the substances from the experimental reactor system.

**Conclusion:**

Our study demonstrates that a number of chemical messengers, agonists and antagonists induce contraction and/or modulate the endogenous contraction rhythm and amplitude of our nerveless model metazoon *T. wilhelma*. We conclude that a relatively complex system of chemical messengers regulates the contraction behaviour through auto- and paracrine signalling, which is presented in a hypothetical model. We assume that adrenergic, adenosynergic and glycinergic pathways, as well as pathways based on NO and extracellular cAMP are candidates for the regulation and timing of the endogenous contraction rhythm within pacemaker cells, while GABA, glutamate and serotonine are candidates for the direct coordination of the contractile cells.

## Background

Sponges are nerveless and muscleless multicellular animals, which emerged early during the evolution of the metazoa [1]. Therefore, sponges represent valuable model systems to conclude upon a hypothetical Urmetazoa, from which the metazoan bodyplan evolved [2-4]. Despite the fact, that sponges do not posses muscles and a nervous system, they are able to react upon external stimuli [5-7], to move [7-10], to contract [summarised in 7], and display diurnal rhythms [7,11]. The earliest description of such sponge behaviour dates back to Aristotle, who mentioned contraction of living sponges, when they were touched and collected by humans for the production of bath sponges [12].

The putative coordination mechanisms have been discussed in details over the last century, with a main focus on the topic in the 1960s and 1970s [8,13-15], which lead to the general acceptance that sponges do not possess a nervous system. However, questions for nervous-system like coordination mechanisms have been raised again from time to time due to new findings, as action potentials in the Hexactinellida [16-18] or the characterisation of a GABA/glutamate-like receptor from *Geodia cydonium *[19].

Jones [8] presented one of the most comprehensive discussions of coordination systems in sponges. As alternative hypothetical signal conduction systems in sponges he named: (a) local fall of pressure inside the canal system, which would be transmitted through the system to induce contraction in distant parts of the sponge; (b) mechanical conduction of contraction forces through the interconnected contractible sponge tissue (presumably the continuous pinacoderm); (c) a chemical messenger, distributed by the aquiferous system; (d) a chemical messenger diffusing through the mesohyle; (e) distribution of action potentials by intercellular junctions. Even though Jones seems to prefer the mechanical mechanism based hypotheses, he states that "clearly the mechanism cannot be a simple one" (p. 41). However, though mechanical conduction may explain the transmission of a physical information, it can not explain how endogenous rhythms are generated and coordinated. This is especially of importance concerning the diurnal patterns found in sponges [7,11]. In this context, chemical messenger based systems, acting via specific ligand-receptor interactions, seem to be more likely. In fact, a number of transmitters, chemical messengers, receptor agonists and antagonist have been tested on sponges. The first experiments date back to Lendenfeld at the End of the 19^th ^century [20]. They were followed by others, partly with contradictory results, e.g. positive reports upon acetylcholine in sponges by some investigators [21,22] and negative reports by others [5,23-25]. However, this inconsistencies of results can be partly explained by technical reasons and the fact that varying markers, sponge species and sponge reactions (contraction, internal current, state of the oscule) were regarded. In addition, some of these experiments suffered from the lack of precise quantitative measurement methods.

**Figure 1:**
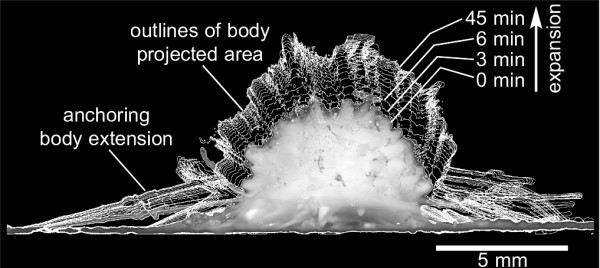
**Expanding specimen of *Tethya wilhelma ***Overlay of a time-lapse series of outlines of the projected body area of *T. wilhelma*, representing an expansion from fully contracted state at t = 0 min to fully expanded state at t = 45 min. In addition, the 8-bit image of the contracted sponge body at t = 0 min is displayed on top of the overlay, to give an impression of the sponge and it's body extensions. Like the body outlines, the measurement of the projected area is computed automatically using the software ImageJ 57 and own macro functions. Outline and overlay were computed using ImageJ, too.

To overcome these problems, we have established a model system, based on the contractile and locomotive sponge *Tethya wilhelma *(Demospongiae, Hadromerida; Fig. 1), which has been described from an aquarium habitat recently and can be maintained and reared in the aquarium [7,26-30]. The use of digital time lapse imaging of the sponge body, in conjunction with quantitative image analysis, allows us to record and analyse the behaviour and reactions of the sponge quantitatively by measuring changes of the projected body area (for the principle see in Fig. 1). Using this methodology, one of us recently demonstrated that *T. wilhelma *displays a regular endogenous contraction rhythm, which is diurnal and can be disturbed by external events. This integrative behaviour characterises *T. wilhelma *as a valuable model system for research on coordination in basal metazoa [7]. Furthermore, we have shown that the contraction of T. wilhelma is inducible by chemical messengers and we characterised the specificity, dose response and kinetics of contraction for the neuroactive amino acids γ-amino butyric acid (GABA) and glutamate [31], giving evidence for the presence of a GABAergic coordination mechanism in sponges.

**Figure 2:**
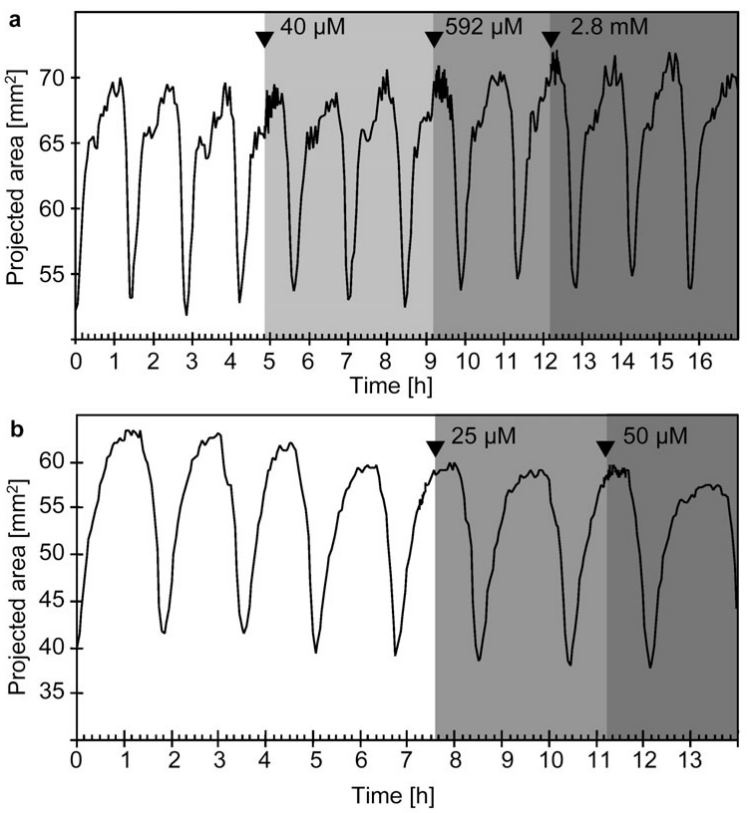
**Contraction patterns of *Tethya wilhelma *during extracorporeal acetylcholine and nicotine application **(a - b): Projected area of two specimens of *T. wilhelma *before and during application (grey background) of acetylcholine (a) and nicotine (b) respectively. Neither of the two substances induced contraction. See additional file 1 (Movie_S1.mov) for the time-lapse movie of Fig. 2b. Filled arrowheads indicate the points in time when stock solutions of the substances were injected into the experimental reactor circulation system to reach final concentrations of 40 μM, 592 μM and 2.8 mM for acetylcholine and 25 μM and 50 μM for nicotine.

In the present study we evaluated the effects of a number of substances upon our model sponge, by means of induction of contraction and interference with endogenous contraction rhythm and amplitude. We have chosen substances acting via ligand-receptor systems, which are known to be involved in the regulation of behaviours of metazoa with central nervous systems (CNS): acetylcholine and nicotine, caffeine, glycine, adrenaline, serotonine, nitric oxide and cyclic AMP. Most of these substances have been previously tested in various sponges under varying conditions and set-ups by various sponge researchers as shown above. In contrast, we used a semi-closed experimental reactor, under reproducible experimental conditions. The aim of the present study was to demonstrate that contraction, endogenous rhythm and strength of contraction are specifically modulated by several neuroactive chemical substances. Consequently, we aimed at adding more details to our hypothetical chemical messenger-based coordination model, presented before [31]. In addition, the present study should help us to identify ligand-receptor system candidates, which deserve more detailed physiological and molecular characterisation in *T. wilhelma *and eventually other sponge models.

## Results

### Acetylcholine and nicotine

For acetylcholine, we tested concentrations between 40 μM and 2.8 mM (Fig. 1a), for nicotine, we tested concentrations from 25 μM to 50 μM (Fig 1b). Neither acetylcholine, nor nicotine induced contractions or altered the endogenous contraction rhythm or amplitude on first sight. In both cases, the endogenous contractions are rhythmical and equally strong, running as waves over the body of the sponge from the basal attachment area to the apical part of *Tethya *(see Additional file 1: Movie_S1.mov). However, comparing contraction cycle durations (D_cc_) statistically, revealed a slightly but significantly prolonged cycle duration for acetylcholine exposure, whereas no significant difference was found for nicotine (Tab. 1).

### Caffeine

**Figure 3:**
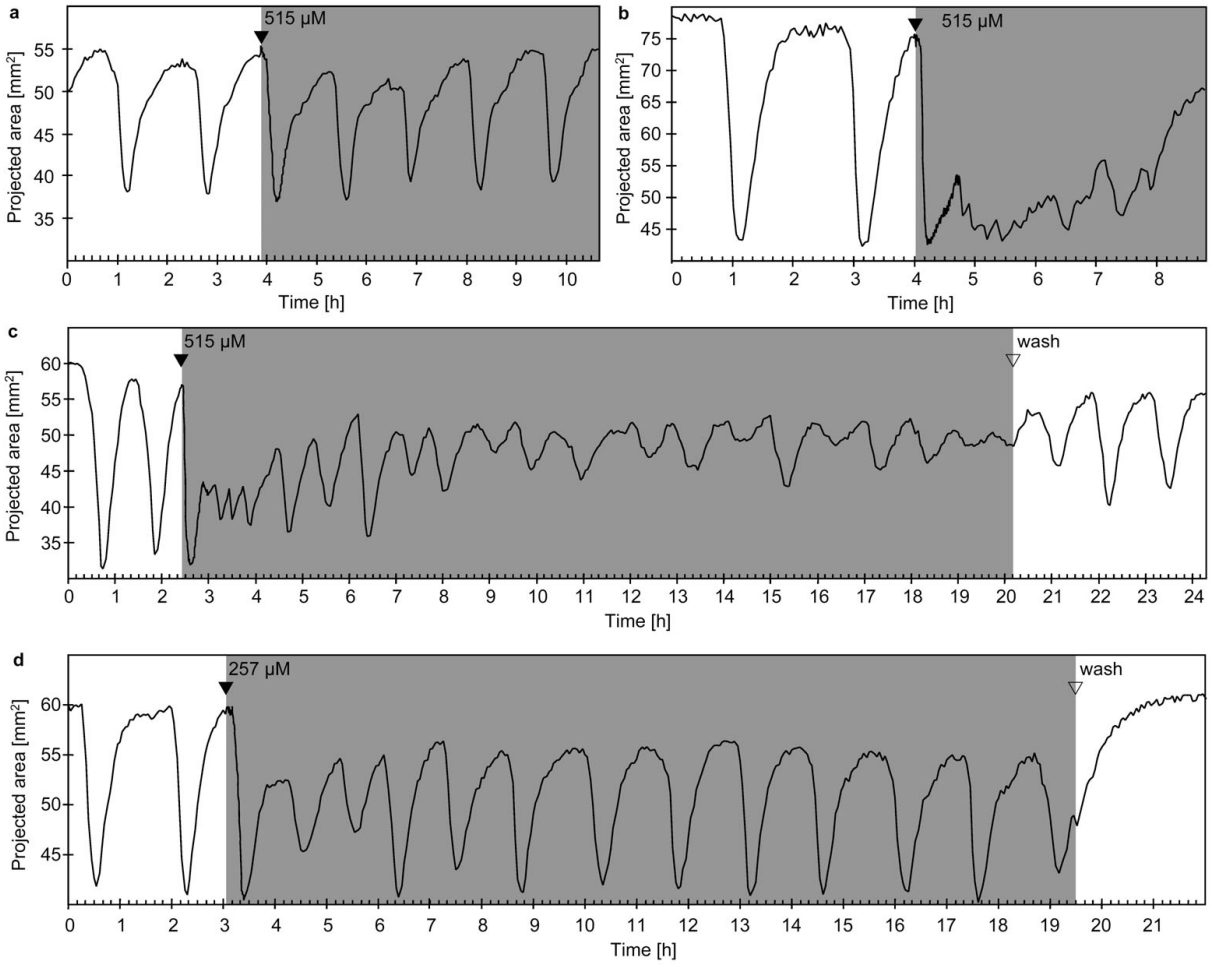
**Contraction patterns of *Tethya wilhelma*during extracorporeal caffeine application **(a - d): Projected area of four specimens of *T. wilhelma *before, during (grey background) and after caffeine application. Caffeine induced contractions in all experiments. A spasm-like pattern representing an extremely prolonged phase in contracted state was observed once (b; see additional file 2: Movie_S2.mov for the time-lapse movie of Fig. 3b). Long-term application resulted in an irregular contraction pattern in some cases (c, d; see additional file 3: Movie_S3.mov for the time-lapse movie of Fig. 3c). Filled arrowheads indicate the points in time when stock solutions of the substances were injected into the experimental reactor circulation system to reach final concentrations of 515 μM (a - c) and 257 μM (d) respectively. Open arrowheads indicate the wash out of the substance from the experimental reactor system.

In all experiments, caffeine induced contractions at concentrations of 257 μM and 515 μM respectively. In contrast to the previously reported almost immediate induction by glutamate and GABA [31], the caffeine-induced contraction is delayed by several minutes in most cases (Fig 3a, b, d), though immediate onset may occur, too (Fig. 3b). In one case, we found a spasm-like reaction of *T. wilhelma *upon caffeine exposure. This spasm resembles very much the spasms, which can be induced by GABA [31]. After regular contractions and expansions the sponge is stimulated by caffeine, contracts and remains in an almost contracted state, with a fast progression of minor expansions and contractions (Fig. 3b). The time-lapse recording shows that the sponge tissue does not contract in a temporally coordinated way (see Additional file 2: Movie_S2.mov). Local contractions and expansions of parts of the sponge tissue occur concurrently, shaking the sponge in a spasm-like manner.

In addition, caffeine triggered long-term effects by altering both, D_cc _and the strength of the overall contraction. The D_cc _is shortened significantly during long-term caffeine exposure (Tab. 1), but the effect is suspended by washout of caffeine from the experimental reactor. The same applies for the alterations of the overall contraction strength during caffeine-exposure. The amplitude of the recorded changes in projected body area is attenuated (Fig. 3c, d). In the most prominent case *T. wilhelma *reached a semi-contracted state after several hours and displayed only weak contractions (Fig. 3c). The time-lapse recording reveals a behaviour of the sponge tissue similar to the spasm-like state, but the effect is weaker and the endogenous rhythm continues (see Additional file 3: Movie_S3.mov).

### Glycine

**Figure 4:**
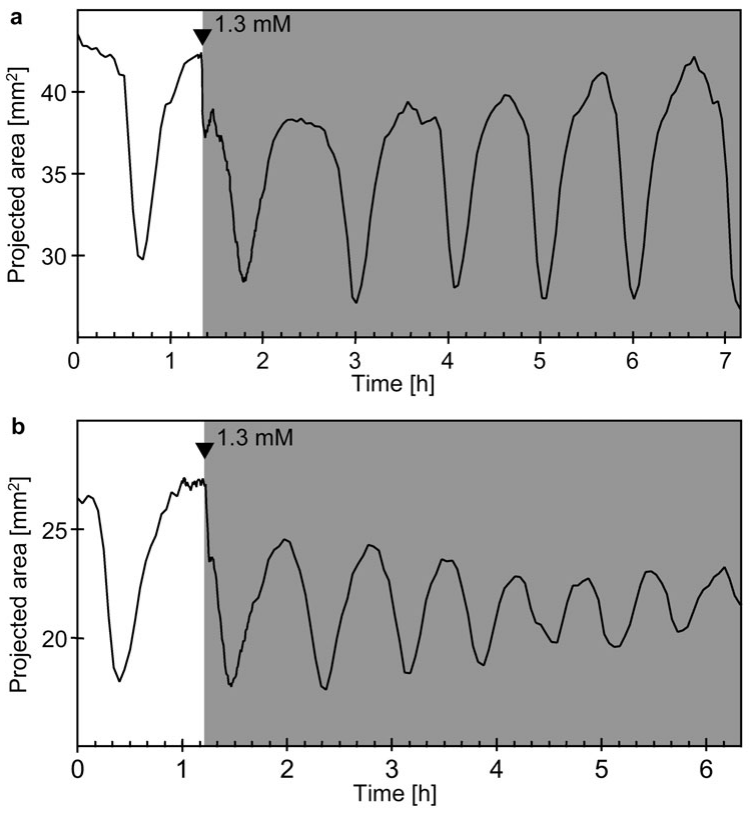
**Contraction patterns of *Tethya wilhelma *during extracorporeal glycine application **(a - b): Projected area of two specimens of *T. wilhelma *before and during glycine application (grey background). Glycine induced bi-phasic contractions in all experiments and attenuated the contraction amplitude (b; see additional file 4: Movie_S4.mov for the time-lapse movie of Fig. 4b). Filled arrowheads indicate the points in time when stock solutions of the substances were injected into the experimental reactor circulation system to reach final concentrations of 1.3 mM.

Like caffeine, glycine induced contraction and altered D_cc _and the overall strength of contraction. The induced contraction is characterised by a bi-phasic appearance (Fig. 4). Time-lapse recordings reveal that in the first phase, the sponge contracts only in the basal part of the body. Consequently, the sponge drops towards the substrate and the tissue of the whole body starts to contract quite simultaneously in the following second phase (see Additional file 4: Movie_S4.mov, time index 69 – 90 min).

The endogenous D_cc _is significantly shortened during glycine exposure (Tab. 1), but restores after wash out of the substance. The amplitude of the recorded changes in projected body area may be attenuated (Fig. 4b). The attenuation differs from the one observed for caffeine, since the overall appearance of the contraction is very similar to the non-induced endogenous contractions, except that the volume changes during contraction and expansion are lower (see Additional file 4: Movie_S4.mov, time index 90 – 378 min).

### Adrenaline and serotonine

**Figure 5:**
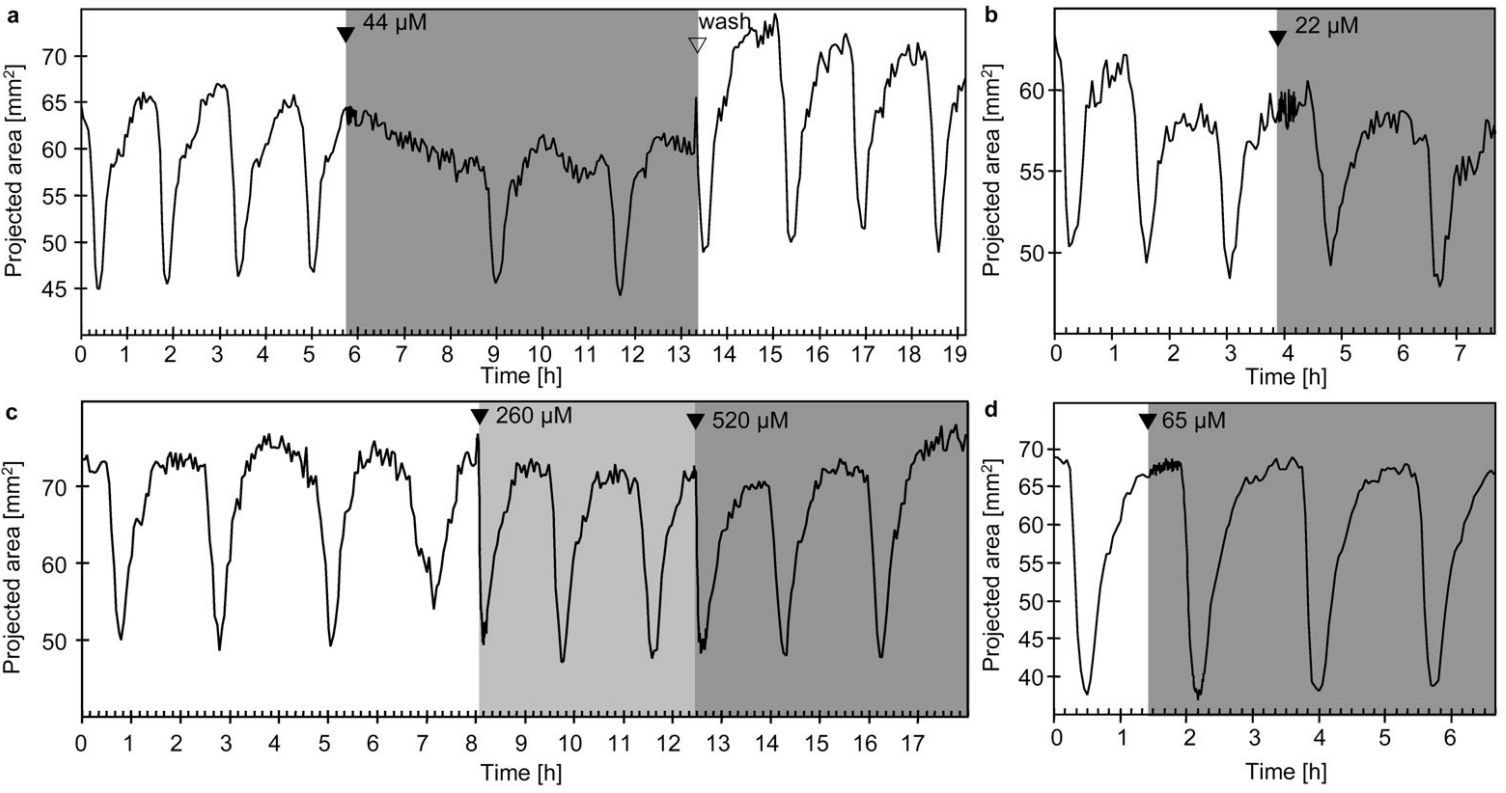
**Contraction patterns of *Tethya wilhelma*during extracorporeal adrenaline and serotonine application **(a - d): Projected area of four specimens of *T. wilhelma *before, during (grey background) and after application of adrenaline (a - b) and serotonine (c - d) respectively. Adrenaline did not induce contractions, but resulted in prolonged phases of expansion (a, b; see additional file 5: Movie_S5.mov for the time-lapse movie of Fig. 5c). Serotonine induced contractions at higher concentrations (c), but not at lower concentrations (d). Filled arrowheads indicate the points in time when stock solutions of the substances were injected into the experimental reactor circulation system to reach final concentrations of 44 μM (a) and 22 μM (b) for adrenaline and 65 μM (d), 260 μM and 520 μM (c) for serotonine. Open arrowheads indicate the wash out of the substance from the experimental reactor system.

Adrenaline did not induce contraction in *T. wilhelma*. Nevertheless, the endogenous rhythm is strongly disturbed by adrenaline, leading to longer irregular D_cc _during exposure (Fig. 5a, Tab. 1). The time-lapse recording shows that *T. wilhelma *remains in the expanded phase over a longer period. No local contractions on the surface are observable. Elongation and retraction of body extensions as well as bud-formation are not affected by adrenaline (see Additional file 5: Movie_S5.mov). We found that the effect on D_cc _was stronger at 44 μM than at 22 μM adrenaline (Fig. 5b).

In contrast, serotonine immediately induced contractions at concentrations of 260 μM and 520 μM, but not at 65 μM (Fig. 5c, d). However, we found no attenuation of the strength of contraction and no significant alteration of the D_cc _(Tab. 1).

### Nitric oxide

**Figure 6:**
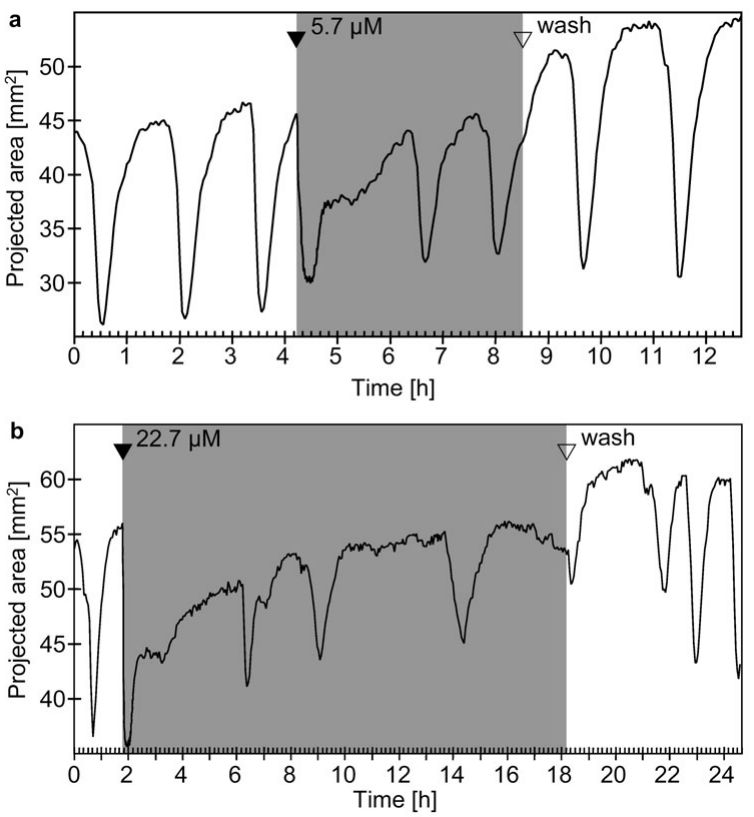
**Contraction patterns of *Tethya wilhelma*during extracorporeal nitric oxide application (via release by NOC-12) **(a - b): Projected area of two specimens of *T. wilhelma *before and during NOC-12 application (grey background). The nitric oxide released by NOC-12 induced immediate contractions in all experiments, attenuated the contraction amplitude and disturbed the rhythm of endogenous contractions (see additional file 6: Movie_S6.mov for the time-lapse movie of Fig. 6b). Filled arrowheads indicate the points in time when stock solutions of the substances were injected into the experimental reactor circulation system to reach final concentrations of 5.7 μM (a) and 22.7 μM (b) for NOC-12.

The NO-releasing substance NOC-12 was found to induce contractions instantly after application (Fig. 6). In addition, strong effects, both on D_cc _and the strength of contraction were recorded. The D_cc _is significantly increased and appears to be more irregular (Tab. 1, Fig. 6b). The contraction strength is lowered, with lower volume at expanded phase and higher volume at contracted phase. During this semi-expanded periods, *T. wilhelma *displays almost no local contraction activity (see Additional file 6: Movie_S6.mov). The contractions, which occur within the time of exposure to NO, are similar to non-induced endogenous contractions, except of the attenuated amplitude: they seem to be triggered locally and spread over the sponge body in a wave like manner.

### Cyclic AMP

**Figure 7:**
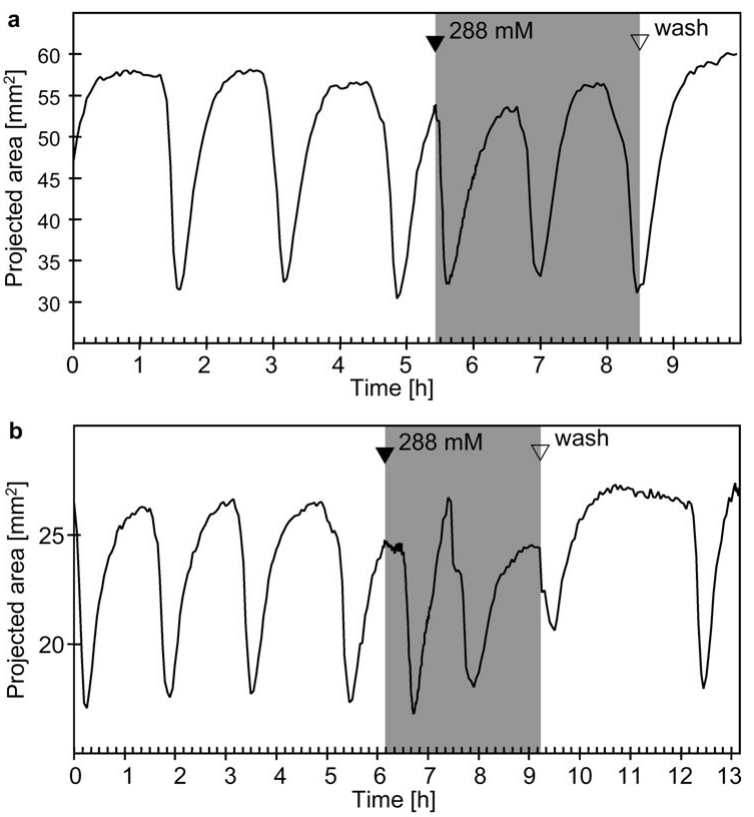
**Contraction patterns of *Tethya wilhelma*during extracorporeal cAMP application **(a - b): Projected area of two specimens of *T. wilhelma *before and during cAMP application (grey background). Cyclic AMP induced immediate (a) and delayed (b) contractions and slightly disturbed the rhythm of endogenous contractions (see additional file 7: Movie_S7.mov for the time-lapse movie of Fig. 7b). Filled arrowheads indicate the points in time when stock solutions of the substances were injected into the experimental reactor circulation system to reach final concentrations of 288 mM for NOC-12.

The application of cAMP resulted in contractions, either with immediate onset (Fig. 7a) or delayed onset (Fig. 7b). In either case there is a tendency for an altered D_cc _(Tab. 1) and the typical pattern was slightly disturbed, as displayed in one case by an immediate second contraction after the initial one, without a typical rest in the expanded state (Fig. 7b). Even though the rhythm is slightly disturbed, the overall appearance of the contractions is not altered during application of cAMP. Like in endogenous contractions, it seems to be triggered locally in the basal part of the sponge and to spread from there towards the apical part (see Additional file 6: Movie_S6.mov).

### General observations and control experiments

In general, most of the sponges responded to the washout of the substances, too. In some cases relatively strong contractions occurred (Fig. 5a; 6b; 7c), in others, *T. wilhelma *responded only slightly (Fig. 3d; 6a). In all cases, water temperature and other physical characters of the replacing artificial sea water were as close as possible to the conditions in the chamber. In almost all cases, the sponge expanded to a higher volume after a wash in comparison to the expanded volume during the experiment (Fig. 3c, d; 7a, b) and in some cases even higher than prior to the experiment (Fig. 5a; 6a, b). The control experiments show, that injection of small volumes of seawater alone into the system does not result in contractions. This is also reflected by those substances, which do not induce contraction, like acetylcholine and nicotine (Fig. 3) or adrenaline (Fig. 5a, b). The pH value of the seawater inside the experimental reactor is slightly altered by some of the substances. Differences of the pH (Δ pH) higher than ± 0.15 were observed only for cAMP (Δ pH = -1.16 ± 0.02; N = 3). An analogous pH change, imitated by applying a corresponding amount of HCl to *T. wilhelma *did not result in contractions (data not shown). Temperature changes within the range of ± 0.5°C, which occurred during the experiments did not affect the sponges in control experiments. The oxygen concentration was kept on a saturated level throughout all experiments, to exclude any influence.

## Discussion

### Effects of neuroactive substances on sponges

We applied substances, which are well-known to be neuroactive in metazoans with a CNS, to our nerveless model metazoon, the sponge *Tethya wilhelma*. The sponge responded by contractions as well as altered rhythm and/or attenuated contraction amplitude to a number of these substances. The results are summarized in Table 2 and compared to previous reports concerning the Porifera.

**Table 1:** Statistical analysis of contraction cycle durations in *Tethya wilhelma *Results of a Mann-Whitney U-test on differences between endogenous and influenced contraction cycle durations (D_cc_) of *T. wilhelma *(each comparison within one experimental series).

**Substance**	**Concentration**	**Figure**	**D**_cc _**endogenous [min]**	**D**_cc _**substance-exposed [min]**	**U-test**	**significant difference in Dcc?**
Acetycholine	2.8 mM	2a	78.3 ± 5.3 (N = 9)	86.9 ± 2.3 (N = 9)	p < 0.001	yes
Nicotine	25 – 50 μM	2b	101.3 ± 8.6 (N = 4)	111.3 ± 10.6 (N = 4)	p > 0.01	no
Caffeine	515 μM	3a	93.1 ± 7.6 (N = 6)	85.3 ± 7.3 (N = 6)	p > 0.01	no
	515 μM	3b	105.0 ± 7.7 (N = 5)	44.12 ± 21.0 (N = 5)	p < 0.01	yes
	515 μM	3c	85.5 ± 19.3 (N = 14)	52.5 ± 17.2 (N = 18)	p < 0.001	yes
	257 μM	3d	113.3 ± 34.6 (N = 9)	78.8 ± 23.5 (N =)	p < 0.01	yes
Glycin	1.3 mM	4a	105.5 ± 23.5 (N = 6)	54.2 ± 9.1 (N = 14)	p < 0.001	yes
	1.3 mM	4b	93.0 ± 10.7 (N = 9)	42.7 ± 5.5 (N = 10)	p < 0.001	yes
Adrenaline	44 μM	5a	88.7 ± 3.4 (N = 7)	154.8 ± 58.7 (N = 4)	p < 0.01	yes
	22 μM	5b	88.5 ± 7.9 (N = 8)	109.8 ± 6.0 (N = 2)	n.d.^a^	n.d.^a^
Serotonine	260 – 520 μM	5d	115.3 ± 14.0 (N = 5)	96.3 ± 10.0 (N = 5)	p > 0.01	no
	65 μM	5c	105.0 ± 8.8 (N = 5)	102.9 ± 2.8 (N = 4)	p > 0.01	no
(by NOC-12)	5.7 μM	6a	75.8 ± 13.0 (N = 8)	116.0 ± 23.8 (N = 8)	p < 0.01	yes
	22.7 μM	6b	95.0 ± 9.4 (N = 6)	238.0 ± 58.2 (N = 5)	p < 0.01	yes
cAMP	288 mM	7a	108.0 ± 11.8 (N = 4)	83.3 ± 5.3 (N = 2)	n.d.^a^	n.d.^a^
	288 mM	7b	105.5 ± 23.5 (N = 6)	73.5 ± 3.5 (N = 2)	n.d.^a^	n.d.^a^

^a ^not determined, not enough data available

**Table 2:** Summary of induced effects on *Tethya wilhelma*Overview regarding induced contraction, endogenous contraction rhythm alteration and/or amplitude attenuation by substances used in the present and previous study, including effects on sponges, reported by other authors before.

**Substance**	**Contraction induced**	**Rhythm altered**	**Amplitude altered**	**Signalling pathway known to be influenced**	**Other reports on sponges**
Acetycholine	-	slightly slower rhythm	-	ionotrophic or metabotrophic acetylcholine receptor	Increased rhythm and intensity of contraction in *Euspongia officinalis *[22], no effect in *Cliona celata *[5]. Acetylcholine esterase activity in *Sycon *sp. 21, *Hippospongia *[35] and *T. wilhelma *[26]
Nicotine	-	-	-	agonist at ionotrophic acetylcholine receptor	Loss of current at high (cytotoxic) concentrations in *Cliona celata *[5]
Caffeine	X^a^	faster rhythm	attenuated	antagonist at metabotrphic adenosine receptors	-
GABA^b^	X	n.d.	-	ionotrophic or metabotrophic GABA receptors	No effect in *Cliona celata *[5]. GABA/mgluR-like receptor gene in *Geodia cydonium *[19]. High specificity in inducing contractions in *T. wilhelma *(Ellwanger et al., submitted).
Glutamate^b^	X	n.d.	-	metabotrophic glutamate receptors	Induces intracellular Ca2+ increase. GABA/mGluR-like receptor gene in *Geodia cydonium *[19]. Lower specificity than GABA in inducing contractions in *T. wilhelma *[31]
Glycin	X	faster rhythm	attenuated	metabotrophic glycin receptors	-
Adrenaline	-	very slow, irregular rhythm	-	metabotrophic adrenergic receptor family	Slight current reduction in *Cliona celata *[5]. Histochemical detection in *Sycon *sp. [21]; increased rhythm and intensity of contraction in *Euspongia officinalis *[22]
Serotonine	X	-	-	ionotrophic or metabotrophic serotonine receptors	No effect in *Cliona celata *[5]. Histochemical detection in *Sycon *sp. [21], and *Tedania ignis *[42]
(by NOC-12)^c^	X	very slow, irregular rhythm	attenuated	intracellular soluble guanylate cyclase	NOS activity in heat stress response of *Axinella polypoides *and *Petrosia ficiformis *[48]
cAMP^d^	X	tendency for slower, irregular rhythm	-	metabotrophic cAMP receptor	Reduced locomotion an altered shape in dissociated *Clathrina cerebrum *cells [51]; Release of *Spongilla lacustris *gemmules from dormancy [50]

^a ^delayed contraction

^b ^detailed data and results presented elswhere [31]

^c ^extracellular application, membrane permeable

^d ^extracellular application, membrane impermeable

Acetylcholine (ACh) is one of the best investigated transmitters and the related muscarinic and nicotinic receptors were the first for which the pharmacology was elucidated [32]. The nicotinic receptors are ligand-gated ion channels, the muscarinic types are metabotrophic systems. Both play important roles in the CNS of all phyla, in which they have been investigated [33,34]. Specific acetylcholine esterase (AChE) activity has been reported for the sponges *Sycon *sp. [21], *Hippospongia communis *[35]. and also for *T. wilhelma *[26]. On the other hand, no AChE activity was found by investigators in the first half of the 20th century, though several sponge specimens have been investigated: *Spongilla lacustris *[23], *Scypha *sp. [24], *Leuconia asperta *and *Syphonocalina crassa *[25]. On the other hand, according to Pavans de Ceccatty [22], the application of ACh increases the rhythm and intensity of contraction in *H. communis*. In contrast to this results and our own previous finding of AChE activity in *T. wilhelma*, our present investigations do not reveal a significant correlation between ACh and contraction. ACh does not induce contraction. Nevertheless, the slight, but statistically significant effect of ACh on contraction cycle duration provides evidence that ACh plays a role in *T. wilhelma*, but is not directly involved in the coordination of contraction. However, it may be involved in the regulation of body extension formation and retraction [26], which has to be proven. Our present results show that nicotine does not affect contraction and rhythm in any way. Therefore, we can conclude that at least ionotrophic ACh-receptors are most likely not involved in regulation of contraction in *T. wilhelma*.

Caffeine is a non-specific antagonist of adenosine-receptors in the mammalian brain [36]. In addition, as stated by Fredholm and co-authors, it is the „most widely consumed behaviourally active substance in the world" [36]. In this context, it is strange, that to our knowledge, no coffee-consuming sponge scientists has ever reported any caffeine tests upon sponges. Our own tests on *T. wilhelma *display a strong effect: it induces contractions, increases the endogenous rhythm and attenuates the amplitude of endogenous contractions. Interestingly, in most experiments, the onset of caffeine-induced contraction is decelerated. Except for cAMP (see below), we have not observed such a pattern in any other contraction-inducing substance. Consequently, we conclude from the decelerated onset, that caffeine does not act directly upon the contractile cells. From the disturbed endogenous rhythm and contraction amplitude, it seems likely that caffeine interferes with the regulation of endogenous contractions. The spasm-like reaction (Fig. 3b) and the chronotropic effect, viz. the significantly faster, though slightly irregular contraction rhythm, underline this hypothesis. Consequently, a putative adenosine receptor may be part of the triggering mechanism of endogenous contractions. In mammals, adenosine receptors are ubiquitous and important throughout all kind of tissues and cell types and they have been shown to cause chronotropic effects in the heart [review in 37]. In addition, adenosine receptors, which are all metabotrophic, link the ATP-driven energy metabolism, to the signalling pathways of the cells. The adenosine signalling pathway strongly interacts with other messenger substances [38]. It is very likely that such a central signalling mechanism evolved early and plays an important role in invertebrates, too [33].

Glycine induces effects similar to caffeine in *T. wilhelma*: it stimulates contraction, fastens the rhythm of contraction, while attenuating the amplitude. Beside GABA and glutamate, for which we have characterised the effects on *T. wilhelma *in details elsewhere [31], glycine is one of the important amino acid transmitter in the vertebrate brain [39]. Its transmitter function has also been demonstrated in invertebrates, e.g. *Hydra vulgaris*, where it acts as an inhibitory transmitter, too [40]. In our experiments *T. wilhelma *showed a biphasic response, with a characteristic initial dropping of the sponge towards the substrate, followed by the decelerated body contraction. This indicates that glycine most likely acts upon cells which are involved in the regulation of endogenous contractions, not upon the contractile cells directly. This is underlined by the altered rhythm and amplitude. It has been demonstrated before that amino acids play a role in the intercellular signalling of sponges:. Recently, we characterised the kinetics of GABA- and glutamate-induced contractions in *T. wilhelma *[31]. In relation with the finding of a GABA/glutamate-like receptor gene in *Geodia cydonium *[19] and the observation of a slower current under GABA exposure for *Cliona celata *[5], we conclude that the GABAergic signalling pathway exists in sponges. Our first indications for a glycinergic system based on the results of the present study will have to proven by further molecular and biochemical investigations.

The catecholamine adrenaline is another substance, which has been tested on sponges previously: by Emson on *Cliona celata*, observing a slight reduction in current [5], and by Lentz who specifically stained adrenaline-containing bipolar and multipolar cell types in *Sycon *sp., using histochemical methods [21]. The result of our present study underlines that adrenaline plays a role in sponges. The strict limitation of effects to the elongation of endogenous contraction cycle duration in *T. wilhelma *suggests that adrenalin is directly involved or alt least interfering with the regulation of the endogenous contraction rhythm. In mammals, adrenaline is involved in the upregulation of genes of the molecular clock and is therefore involved in the feedback loop regulation of circadian rhythm [41]. In this context, it is interesting that Reiswig and Nickel reported in independent publications on circadian rhythms in two different *Tethya *species [7,11]. In conclusion, adrenaline might directly be involved in the circadian regulation in *Tethya*.

The effect of the biogenic amine serotonine upon sponges has also been tested previously. It has been detected twice using histochemical and imunohistochemical methods in *Sycon *sp. and *Tedania ignis *[21,42]. However, Emson found no effect upon *Cliona celata *[5]. In contrast, serotonine did immediately induce contraction in *T. wilhelma *in the present study at concentrations of 260 μM and higher. The effect is limited, since the rhythm of endogenous contractions is not affected. However, the widespread finding of serotonine in all phyla of the metazoa, acting via ionotrophic and metabotrophic receptor types, points to an early evolution of this central signalling system [33,34]. This is also supported by molecular data [43].

The gas nitric oxide (NO) is a central molecule in the regulation of a high number of physiological processes in vertebrates [44], but NO also plays a role in many invertebrate phyla [45,46]. It acts upon soluble guanylate cyclase and is released by nitric oxide synthase (NOS) during conversion of l-arginine to l-citruline. This reaction is mainly regulated by intracellular Ca^2+^. NO diffuses directly from cell to cell and its range is limited by its short half life [47]. NO is supposed to be an evolutionary old messenger, which acts auto- and paracrine [33]. This is supported by the finding of NOS in the sponge *Axinella polypoides*, where it is involved in the temperature signalling cascade [48]. Here we showed that NO induces contraction in *T. wilhelma*, but also modulates the endogenous contraction rhythm and amplitude. Due to its fast diffusion, the short half life and the limited range, NO is an optimal candidate for a contraction-inducing messenger, which acts auto- and paracrine and my also be involved in a feedback loop to putative triggering cells, involved in the timing of the endogenous rhythm.

Cyclic AMP also induced contractions in *T. wilhelma *in the present study. It is of interest that we did not use a membrane permeable form of cAMP. Consequently, the observed effect has to be regarded as a specific extracellular signalling event, which is most likely modulated by a specific cAMP receptor, similar to *Dictyostelium discoideum *[49]. It has been shown before that extracellular cAMP plays a regulative roll in Porifera: it is involved in the regulation of production and development of gemmules in freshwater sponges [50], and it directly affects the locomotion of dissociated cells of *Clathrina cerebrum *[51]. It may therefore link cellular mobility and body locomotion with contraction in sponges. All three phenomena are important aspects in the life of *Tethya *[7,9,10,26,52].

### Ligand-based coordination pathways in sponges

**Figure 8:**
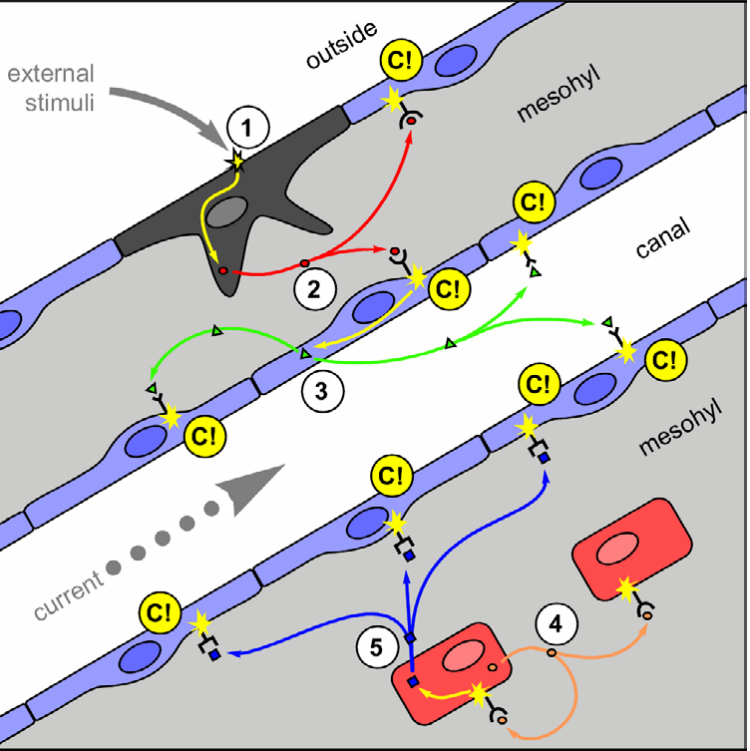
**Hypothetical signalling pathways in *Tethya wilhelma *involved in coordination of contractions upon external stimuli and endogenous signals **An external stimulus (1) at a putative receptor cell (grey) in the pinacoderm triggers the release of a signal substance (2) which diffuses through the mesohyle (light blue) of the sponge and triggers the contraction (C!) of contractile pinacocytes (blue) via a specific receptor and an intracellular signalling pathway. Eventually, stimulated pinacocytes release a second signal substance (3), which may further diffuse through the mesohyle or be distributed by currents in the canal system. Such a secondary signal would amplify the reaction speed upon external and internal triggering of contraction. The endogenous contraction rhythm may be controlled by numerous trigger cells (red) distributed in the mesohyle. These cells are supposed to release an auto-/paracrine signal substance (4), which diffuses through the mesohyle to coordinate the release of a signal substance (5), which diffuses to the pinacocytes and triggers contraction and eventually results in a signal amplification like shown in step (3). Signal substances (2) and (5) are likely not identical to allow independent specific coordination of contraction upon endo- and exogenous stimuli. See Additional file 8: Movie_S8.mov for a stepwise presentation of the hypothetical model.

The question whether sponges possess a nervous (-like) system or not, has been discussed in detail and is generally answered in the negative [4], for an overview see [7,8,14,15]. Even the reports of action potentials in the Hexactinellida did not alter this conclusion, since a syncytial tissue is involved in this there [16-18]. The most detailed discussion is given by Jones [8], who also discussed alternative coordination mechanisms, based on mechanical signal propagation, which he preferred to chemical messenger based mechanisms. However, our present reports of specific induction of body contraction and the modulation of endogenous contraction rhythm and amplitude by various chemical messengers or receptor agonists and antagonists in our model system *T. wilhelma*, strongly suggests the chemical messenger hypothesis. This hypothesis is supported by or recent detailed kinetic characterisation of the contractile response of *T. wilhelma *upon GABA and glutamate [31], as well as the previous reports on effects of messengers, agonists and antagonists upon sponges (see Tab. 1 and text above). In addition the molecular characterisation of a putative metabotrophic GABA/glutamate-like receptor, clearly shows the presence of messenger based signalling systems in sponges [19]. Consequently, we support the idea of an extensive chemical messenger based signalling system in sponges [8,53]. From our results, the question raises, why so many messenger substances are effective upon a biological system, which obviously has only few behavioural choices? The answer is simple. For a filtering, sessile organism like a sponge the adequate answer to many stimuli or environmental states is to stop filtration and eventually to renew the internal water content of the aquiferous system quickly. Our model system *Tethya wilhelma *most likely alters the body volume by contraction of the pinacoderm of the complex aquiferous system, in order to exchange the water [7,54]. It seems to be favourable for the sponge to exchange the water regularly, which is reflected by the endogenous contraction rhythm. Hence, we conclude upon the presence at least one signalling pathway in conjunction with the regulation an endogenous rhythm. Taking into account that the pacemakers of the endogenous rhythm are most likely not the contractile cells themselves, *Tethya *requires at least two signalling pathways to coordinate the pacemakers via a auto- and paracrine signal and to trigger the contraction of the pinacocytes by a paracrine messenger (Fig. 8).

On the other hand it has been shown that sponges react upon mechanical stimuli [5-7] or changes of external water currents, e.g. during washout events in the present study (see above). Our previous studies on GABA also showed that attenuation and desensitisation mechanisms are part of the regulation systems. Consequently, if contraction would be coordinated by only one signalling system, the sponge would not be able to respond upon further stimuli during periods of attenuation. Therefore we conclude that several paracrine ligand-receptor system based signalling pathways are involved in the coordination of external signals down to the contractile effector cells (Fig. 8). Whether these paracrine signals spread through the mesohyle or if one or more are additionally or even exclusively distributed through the aquiferous system will have to be determined in future investigations.

## Conclusion

Our study demonstrates that a number of chemical messengers, agonists and antagonists induce contraction and/or modulate the endogenous contraction rhythm and amplitude of our nerveless model metazoon, the sponge *Tethya wilhelma*. From our results in combination with previous investigations on signalling and coordination in sponges, we conclude that a relatively complex system of chemical messengers regulates the contraction behaviour through auto- and paracrine signalling, acting upon specific receptor systems. As a consequence of the living conditions of sessile filtering organisms, it seems to be most likely that several different signalling pathways are involved in the coordination of contraction, since this single behaviour is the adequate answer to a number of external stimuli. Furthermore, the present screening on potential messenger systems displays again the value of *T. wilhelma *as a model system to understand the early evolution of signalling systems during the evolution of multicellularity in the basal metazoa. Basically we expect to find the same messenger-receptor-systems, which are part of the nervous systems and paracrine regulation systems of other metazoan phyla. From the present and a previous study, we conclude that an adrenergic, adenosynergic and glycinergic pathways, as well as pathways based on NO and extracellular cAMP are candidates for the regulation and timing of the endogenous contraction rhythm within pacemaker cells, while GABA, glutamate and serotonine are candidates for the direct coordination of the contractile cells. However, most likely these systems act on a different scale and in varied contexts, in comparison to the well-investigated systems in animals with a CNS. This will have to be unravelled by further molecular and physiological characterisations of the signalling systems in *T. wilhelma *and other sponge model systems, e.g. the freshwater sponges.

## Methods

### Sponges

Specimens of the sponge *Tethya wilhelma *Sarà et al. 2001 (Tethyidae, Hadromerida, Demospongiae) were obtained from the type location in the aquarium of the zoological-botanical garden 'Wilhelma' in Stuttgart [27]. For experiments the sponges were maintained in a 180 l aquarium, running at 26°C, using artificial seawater [55], at a light-dark cycle of 12/12 hours. Sponges were fed four to five times a week using suspended commercial invertebrate food (Artificial Plancton, Aquakultur Genzel, Fellbach, Germany, www.aquakultur-genzel.de), by pipetting several ml of suspension to each sponge. Seawater was exchanged at a rate of around 10 % of the total aquarium volume each three to four weeks.

### Experimental reactor

All experimental manipulations were carried out in a 250 ml closed experimental reactor. It consisted of an aerated experimental chamber, designed on the principles of airlift reactors, connected to a temperature regulation unit (F25, Julabo, Seelbach, Germany). Oxygen level and temperature were monitored using a multi-sensor system (P4, WTW, Weilheim, Germany), controlled by a computer-software (MultiLab Pilot 3.0, WTW). A built-in optical glass filter (Ø 49 mm, D.K. Enterprises, India) allowed proper imaging.

### Digital time-lapse imaging and image analysis

Digital images of the sponge specimens were taken at a resolution of 2048 × 1536 pixels at regular intervals of 180 s (pre- and post-experimental monitoring) and 30 s (monitoring for induced contraction), resulting in an image-data accumulation rate of 60 megabytes per hour and 360 megabytes per hour, respectively (uncompressed 8-bit image data). A Nikon Coolpix 990E digital camera in manual macro focus and exposure mode was used to acquire greyscale images. The camera was connected to a Nikon SB 24 flash unit, set to manual mode (24 mm, output 1/16). The camera was controlled by a PC, using USB connection cable and the software DC_RemoteShutter V 2.3.0 in conjunction with DC_TimeTrigger V. 1.0 [56]. Images were downloaded, saved on the PC and erased on the CF-card of the camera instantly after being taken. A reference image including a scale bar placed next to the sponge was taken for each experimental series, to allow scaling. In all cases a black background was used to maximise contrast.

Image analysis was performed using ImageJ 1.30 to 1.34 (NIH, Washington, USA), based on built in functions [57]. The measurement of projected areas bases on the contrast difference between sponge (whitish) and background (black). All images were scaled using the reference image. A threshold value between 50 and 90 was applied to the 8-bit images and the absolute projected area of the sponge was measured using ImageJ's built-in measurement tool. Each time-lapse series was loaded as an image stack into ImageJ. Time-lapse movies were prepared based on built in functions of ImageJ. A macro was programmed to measure semi-automatically. Measurement results were written to a text file and further computed using Excel 2000. We performed Mann-Whitney U-tests on contraction cycle duration (D_cc_) of endogenous contraction periods and substance-exposed periods for single sponge individuals using the Excel add-on WinSTAT [58]. D_cc _was measured as time between two subsequent contractions, defined by body volume minima [7].

### Test substance application

Diluted stock solutions of the test substances were injected into the experimental reactor to reach final concentrations between 5.7 μM and 1.3 mM depending on the substance. For our tests, the following substances were applied: acetylcholine (acytlcholine chloride, Sigma-Aldrich A6625); adrenalin ([± ]-epinephrine, Sigma-Aldrich E1635); cAMP (cyclic adensosine-3'-5'-monophosphoric acid monosodium salt, Boehringer 102296); caffeine (Sigma Aldrich C5-3); glycine (Merck 104201); nicotine ([S]- [-]-nicotine, Sigma-Aldrich 18,637-6); NOC-12 (3-ethyl-3- [ethylaminoethyl]-1-hydroxy-2-oxo-1-triazene, Sigma-Aldrich E3145); serotonin (serotonin creatinine sulfate complex, Sigma-Aldrich H7752).

Care was taken not to inject the solutions directly to the sponge specimen, but allow mixing in the circulating current of the system. For each experiment, substances were only applied during a phase of expansion in-between two subsequent endogenous rhythmic contractions, but the relative time within the contraction cycle varied among the experiment, for image data-processing reasons. The sponges long-term reaction was recorded. After the experiment the reactor was perfused by fresh, aerated artificial seawater at 26°C.The same specimens were eventually used for another experiment after retaining a normal contraction rhythm for at least several hours.

### Control experiments and control measurements

Prior to the experiment, each specimen used was allowed to acclimatise to the experimental reactor for several hours. Experiments were only started after the sponge specimens displayed typical regular contraction patterns as described previously [7]. During all of the experiments, temperature and oxygen-level were monitored and recorded. Changes in pH due to application of substances were measured. Control experiments were carried out to test the sponge's reaction to pH-changes.

## Competing interests

The author(s) declare that they have no competing interest.

## Authors' contributions

KE designed and performed the experiments, analysed the data, prepared figure drafts and revised the manuscript. MN designed the study and principal experiments, performed data analysis, prepared final figures, drafted, wrote and revised the manuscript.

## Supplementary Material

Additional file 1 **Time-lapse movie of *Tethya wilhelma *reacting upon extracorporal nicotine application **The quicktime-movie Movie_S1.mov represents the time-lapse image series of the time period displayed as a contraction pattern graph in Fig. 2b. The time span of extracorporeal application of the substance is indicted in the movie as well as the elapsed time. Time-lapse 2300-fold. For more details refer to the results section of the manuscript. Click here for file

Additional file 2 **Time-lapse movie of *Tethya wilhelma*reacting upon extracorporal caffeine application (spasm-like contraction behaviour) **The quicktime-movie Movie_S2.mov represents the time-lapse image series of the time period displayed as a contraction pattern graph in Fig. 3b. The time span of extracorporeal application of the substance is indicted in the movie as well as the elapsed time. Time-lapse 2300-fold. For more details refer to the results section of the manuscript. Click here for file

Additional file 3 **Time-lapse movie of *Tethya wilhelma*reacting upon extracorporal caffeine application (attenuated amplitude, local contractions) **The quicktime-movie Movie_S3.mov represents the time-lapse image series of the time period displayed as a contraction pattern graph in Fig. 3c. The time span of extracorporeal application of the substance is indicted in the movie as well as the elapsed time. Shifts in perspective are a result of unintentional slight shifts of the camera during the experiment. Time-lapse 2300-fold. For more details refer to the results section of the manuscript. Click here for file

Additional file 4 **Time-lapse movie of *Tethya wilhelma*reacting upon extracorporal glycin application **The quicktime-movie Movie_S4.mov represents the time-lapse image series of the time period displayed as a contraction pattern graph in Fig. 4b. The time span of extracorporeal application of the substance is indicted in the movie as well as the elapsed time. Time-lapse 2300-fold. For more details refer to the results section of the manuscript. Click here for file

Additional file 5 **Time-lapse movie of *Tethya wilhelma*reacting upon extracorporal adrenaline application **The quicktime-movie Movie_S5.mov represents the time-lapse image series of the time period displayed as a contraction pattern graph in Fig. 5a. The time span of extracorporeal application of the substance is indicted in the movie as well as the elapsed time. Shifts in perspective are a result of unintentional slight shifts of the camera during the experiment. Time-lapse 2300-fold. For more details refer to the results section of the manuscript. Click here for file

Additional file 6 **Time-lapse movie of *Tethya wilhelma*reacting upon extracorporal nitric oxide application (via NOC-12) **The quicktime-movie Movie_S6.mov represents the time-lapse image series of the time period displayed as a contraction pattern graph in Fig. 6b. The time span of extracorporeal application of the substance is indicted in the movie as well as the elapsed time. Shifts in perspective are a result of unintentional slight shifts of the camera during the experiment. Time-lapse 2300-fold. For more details refer to the results section of the manuscript. Click here for file

Additional file 7 **Time-lapse movie of *Tethya wilhelma*reacting upon extracorporal cyclic AMP application **The quicktime-movie Movie_S7.mov represents the time-lapse image series of the time period displayed as a contraction pattern graph in Fig. 7b. The time span of extracorporeal application of the substance is indicted in the movie as well as the elapsed time. Shifts in perspective are a result of unintentional slight shifts of the camera during the experiment. Time-lapse 2300-fold. For more details refer to the results section of the manuscript. Click here for file

Additional file 8 **Hypothetical signalling pathways in *Tethya wilhelma*involved in coordination of contractions upon external stimuli and endogenous signals **The quicktime-movie Movie_S7.mov represents a step by step presentation of the hypothetical coordination model given in Figure 8. An external stimulus (1) at putative receptor cell (grey) in the pinacoderm triggers the release of a signal substance (2) which diffuses through the mesohyle (light blue) of the sponge and triggers the contraction (C!) of contractile pinacocytes (blue) via a specific receptor and an intracellular signalling pathway. Eventually, stimulated pinacocytes release a second signal substance (3), which may further diffuse through the mesohyle or be distributed by currents in the canal system. Such a secondary signal would amplify the reaction speed upon external and internal triggering of contraction. The endogenous contraction rhythm may be controlled by numerous trigger cells (red) distributed in the mesohyle. These cells are supposed to release an auto-/paracrine signal substance (4), which diffuses through the mesohyle to coordinate the release of a signal substance (5), which diffuses to the pinacocytes and triggers contraction and eventually results in a signal amplification like shown in step (3). Signal substances (2) and (5) are likely not identical to allow independent specific coordination of contraction upon endo- and exogenous stimuli. Click here for file
